# Serum Levels of Coenzyme Q10 in Patients with Multiple System Atrophy

**DOI:** 10.1371/journal.pone.0147574

**Published:** 2016-01-26

**Authors:** Takashi Kasai, Takahiko Tokuda, Takuma Ohmichi, Ryotaro Ishii, Harutsugu Tatebe, Masanori Nakagawa, Toshiki Mizuno

**Affiliations:** 1 Department of Neurology, Research Institute for Geriatrics, Kyoto Prefectural University of Medicine, Kyoto 602–0841, Japan; 2 AMED-CREST, Japan Agency for Medical Research and Development Kyoto 602–0841, Japan; 3 Department of Molecular Pathobiology of Brain Diseases, Research Institute for Geriatrics, Kyoto Prefectural University of Medicine, Kyoto 602–0841, Japan; 4 Department of Zaitaku (Homecare) Medicine, Kyoto Prefectural University of Medicine, Kyoto 602–0841, Japan; 5 North Medical Center, Kyoto Prefectural University of Medicine, Kyoto 629–2261, Japan; University of Windsor, CANADA

## Abstract

The *COQ2* gene encodes an essential enzyme for biogenesis, coenzyme Q10 (CoQ10). Recessive mutations in this gene have recently been identified in families with multiple system atrophy (MSA). Moreover, specific heterozygous variants in the *COQ2* gene have also been reported to confer susceptibility to sporadic MSA in Japanese cohorts. These findings have suggested the potential usefulness of CoQ10 as a blood-based biomarker for diagnosing MSA. This study measured serum levels of CoQ10 in 18 patients with MSA, 20 patients with Parkinson’s disease and 18 control participants. Although differences in total CoQ10 (i.e., total levels of serum CoQ10 and its reduced form) among the three groups were not significant, total CoQ10 level corrected by serum cholesterol was significantly lower in the MSA group than in the Control group. Our findings suggest that serum CoQ10 can be used as a biomarker in the diagnosis of MSA and to provide supportive evidence for the hypothesis that decreased levels of CoQ10 in brain tissue lead to an increased risk of MSA.

## Introduction

Multiple system atrophy (MSA) is a progressive neurodegenerative disease, clinically characterized by autonomic failure in addition to various combinations of parkinsonism, cerebellar ataxia, and pyramidal dysfunction. The distribution of pathologies classically encompass three functional systems in the central nervous system (CNS): the striatonigral system; the olivopontocerebellar system; and autonomic nuclei of the brainstem and spinal cord in which cytoplasmic aggregates of alpha-synuclein are primarily observed in oligodendroglia [1,2]. However, the pathogenic mechanisms underlying this disease remain unclear, making it difficult to develop effective therapies and diagnostic biomarkers.

The *COQ2* gene encodes an enzyme essential for biogenesis of coenzyme Q10 (CoQ10). Mutations in *COQ2* have recently been found in autosomal-recessive MSA families from Japan [3]. Moreover, screening for *COQ2* polymorphisms in sporadic MSA cases has revealed variants conferring an increased disease risk for MSA in Japanese cohorts [3]. CoQ10, or ubiquinone, is a lipophilic molecule present in cell membranes that functions as an essential cofactor for electron transport in the mitochondrial respiratory chain and as an endogenous antioxidant [4] That discovery prompted a reconsideration of the roles of mitochondrial function and oxidative stress in the pathogenesis of this neurodegenerative disease, and also suggested the potential usefulness of CoQ10 as a blood-based diagnostic biomarker in patients with MSA.

The aim of this study was to assess and compare serum levels of CoQ10 in patients with MSA, patients with Parkinson’s disease (PD) and a control population, and to evaluate whether serum levels of this antioxidant can be used to diagnose MSA.

## Material and Methods

### Ethics statement, subject recruitment, and sample collection

All study subjects provided written informed consent to participate, and the study protocols were approved by the University Ethics Committee (ERB-G-12-1, Kyoto Prefectural University, Kyoto, Japan). Study procedures were designed and performed in accordance with the Declaration of Helsinki. Patients were registered in this institute from April 2008 to August 2014. We enrolled 20 patients with MSA (MSA group) according to the current consensus criteria [5]. We also enrolled 20 patients with PD (PD group) according to the UK Parkinson’s Disease Society Brain Bank criteria [6] and 20 participants with non-neurodegenerative diseases (Control group) as age-matched controls in the same registration. Of note, some participants in the MSA group were diagnosed with “possible MSA” according to the consensus criteria when clinical information and serum samples were obtained. However, we confirmed that all converted to “probable MSA” within a 3-year follow-up period. The modified Rankin Scale (mRS), which is a commonly used scale for measuring dependence [7], was used to grade all participants based on their medical records, because daily activities may affect levels of CoQ10 [2]. Since statins (3-hydroxy-3-methylglutaryl coenzyme A reductase inhibitors) reduce the biosynthesis of CoQ10 in addition to the synthesis of cholesterol [8], we excluded two subjects with MSA and two subjects with non-neurodegenerative diseases who were receiving statin therapy. We thus ultimately analyzed 18 samples from patients with MSA (MSA group), 20 samples from patients with PD (PD group), and 18 samples from participants with non-neurodegenerative disease (Control group). No participants were using medicines or food supplements containing CoQ10. The Control group comprised neurologically normal individuals (n = 1) or patients with various neurological disorders, including demyelinating diseases of the CNS (n = 5), epilepsy (n = 2), brain infarction (n = 3), subdural hematoma (n = 1), myositis (n = 1), normal-pressure hydrocephalus (n = 1), subarachnoid hemorrhage (n = 1), herpes zoster (n = 1), cervical spondylosis (n = 1), and neuropathy (n = 1). We avoided collecting samples for research use alone where possible. Most samples were taken when the participants were required to give blood for routine clinical diagnosis or treatment.

Serum samples were taken through venous puncture and a total of 10 ml of blood was collected in blood collection tubes with clot activator and gel separator (Terumo, Tokyo, Japan). After being allowed to clot for 15 min at room temperature, serum was separated by centrifugation for 10 min at 3000 rpm and distributed in polypropylene vials. Fresh samples obtained from the enrolled subjects were immediately stored at -80°C until used for analysis.

### Measurement of CoQ10

Levels of CoQ10 and its reduced form (CoQ10H_2_) in serum were measured by SRL Inc. (Tokyo, Japan) according to the previously established method using high-performance liquid chromatography [9]. CoQH_2_ is easily oxidized to CoQ10 from the moment of sample extraction. Since precautions were not taken to prevent oxidation of CoQH_2_ at sample collection_,_ we evaluated the total CoQ10 (sum total of CoQ10 and CoQ10H_2_) as an index of CoQ10 deficiency [10]. Corrected CoQ10 levels were further defined by dividing serum CoQ10 levels by total cholesterol levels (T-Cho) (i.e., total CoQ10/T-Chol, CoQH_2_/T-Cho), because cholesterol levels influence CoQ10 levels by forming a conjugated form in blood [11]. Levels of serum cholesterol were enzymatically determined in our clinical laboratory.

### Statistics

The level of significance was set at P<0.05. When data were on a continuous or ordinal scale, the homogeneity of groups was analyzed using the Mann-Whitney U test for comparing two groups and the Kruskal-Wallis for three groups. If the Kruskal-Wallis test yielded significant results, Dunn’s post-hoc test was performed. The Chi-square test was used to evaluate the statistical significance of differences in categorical variables. All analyses were performed using SPSS for Windows version 23 software (IBM Japan Ltd, Tokyo, Japan).

## Results

The demographic characteristics of participants in the MSA, PD and Control groups are summarized in Table 1. We found no significant difference in age or gender among groups: mean ages were 62.3 years in the MSA group, 63.9 years in the PD group, and 61.6 years in the Control group. Female ratios in these groups were 33.3% in the MSA group, 70% in the PD group, and 44.4% in the Control group. Mean disease duration in the MSA group (30.2 months) tended to be slightly, but not significantly, shorter than that in the PD group (67.0 months). The mRS scores did not differ significantly among the three groups: median values were 3 in the MSA group, 2 in the PD group, and 2 in the Control group. The MSA group included no familial cases of MSA, 16 patients (88.9%) with MSA-C and 2 patients (11.1%) with MSA-P (Table 2).

**Table 1:** Characteristics of participants in the MSA, PD and Control groups. Data for continuous variables are expressed as mean ±standard deviation or median (maximum-minimum).

	MSA group (n = 18)	PD group (n = 20)	Control group (n = 18)	*P*
Age (years)	62.3±8.9	63.9±12.1	61.6±11.9	0.635
Female/Male	6/12	14/6	8/10	0.066
Disease duration (months)	30.2±13.5	67.0±85.2	NA	0.633
Hoehn-Yahr stage of PD	NA	3 (1–4)	NA	NA
mRS	3 (1–4)	2 (1–4)	2 (1–3)	0.158

NA: not available

**Table 2:** Concentrations of serum CoQ10 and T-cho in patients with MSA.

Patient	Sex	Age (years)	Clinical phenotype	Disease duration(month)	mRS	CoQ10 (nmol/l)	CoQ10H_2_ (nmol/l)	Total CoQ10 (nmol/l)	T-cho (mg/dl)	Total CoQ10/T-cho ratio
1	M	57	MSA-C	14	2	1052	79	1131	182	6.21
2	M	62	MSA-C	18	2	590	75	665	190	3.50
3	F	66	MSA-C	6	1	477	77	554	203	2.73
4	F	46	MSA-C	24	3	349	49	398	162	2.46
5	F	71	MSA-C	36	2	720	128	848	186	4.56
6	F	71	MSA-C	24	4	450	77	527	221	2.38
7	F	67	MSA-C	36	2	512	71	583	305	1.91
8	F	63	MSA-C	18	1	592	99	691	202	3.42
9	F	45	MSA-P	48	3	402	62	464	174	2.67
10	F	51	MSA-C	48	3	413	63	476	141	3.38
11	M	63	MSA-C	36	3	878	73	951	211	4.51
12	F	50	MSA-P	30	3	544	138	682	242	2.82
13	M	70	MSA-C	26	3	588	131	719	183	3.93
14	F	71	MSA-C	30	2	531	52	583	183	3.19
15	M	67	MSA-C	24	3	373	33	406	191	2.13
16	F	66	MSA-C	24	3	243	13	256	264	0.97
17	F	62	MSA-C	42	3	323	45	368	207	1.78
18	M	73	MSA-C	60	4	336	39	375	171	2.20
				Mean		520.7	72.4	593.2	201.0	3.04
				SD		202.8	34.1	222.6	38.5	1.23

SD: standard deviation

**Fig 1:**
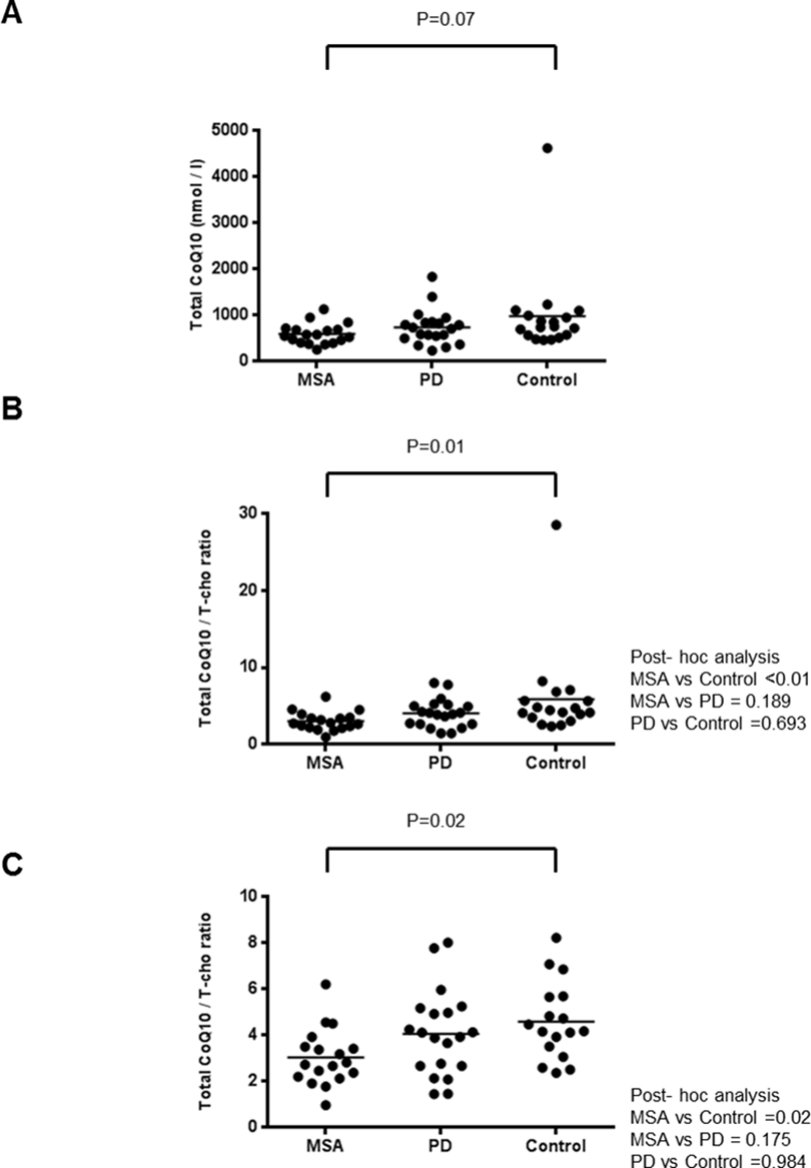
(A) Scatter plot for total CoQ10 level in serum in the MSA group (n = 18), PD group (n = 20), and control group (n = 18). Mean levels of total CoQ10 were lowest in the MSA group (593.2 nmol/l) and highest in the Control group (985.3 nmol/l), although no significant difference was seen between groups. (B) Scatter plot for total CoQ10/T-Cho ratio in serum in the three groups. A significant difference was seen between groups. Post-hoc analysis showed that mean level was significantly lower in the MSA group (3.04) than in the Control group (5.92). (C) The scatter plot for total CoQ10/T-Cho ratio in serum in the three groups after excluding an outlier from the Control group. Similar statistical results to those shown in Graph B were also found. Bars indicate mean values. One participant in the Control group showed an extremely high total CoQ10/T-Cho ratio (Graph B). Graph C therefore shows participants after excluding this outlier. P values over the columns were obtained using the Kruskal-Wallis test. When the Kruskal-Wallis test yielded significant findings, post-hoc analysis was conducted; the results of which are shown on the right side of the graph.

Levels of total CoQ10 are shown in Fig 1A. Mean levels of total CoQ10 were lowest in the MSA group and highest in the Control group. However, comparison of the three groups with the Kruskal-Wallis test showed no significant differences. When we compared corrected levels of total CoQ10 using the ratio of total CoQ10 to T-Cho (total CoQ10/T-Cho) among groups, the ratio was significantly lower in the MSA group than in the Control group (Fig 1B). Mean total CoQ10/T-Cho was higher in the PD group than in the MSA group, although statistical significance was not maintained after post-hoc analysis. An outlier was seen in the Control group, for which we could find no specific factor contributing to the extreme elevation of CoQ10 (e.g., unbalanced diet). To exclude the possibility that the statistical results were unduly influenced by this outlier, we reanalyzed the groups after removing the outlier from the data-set. Exclusion of the outlier made the mean ±standard deviation (SD) values for total CoQ10 and CoQ10/T-cho in the Control group changed from those shown in the Table 2C to 771.1±244.9 and 4.58±1.67, respectively. The significant difference in total CoQ10/T-Cho between the MSA and Control groups was robustly confirmed even after excluding the outlier (Fig 1C). We also examined levels of CoQ10H_2_, which comprised the major part of total CoQ10, although these values were “only advisory”, due to the possibility of sample oxidation. CoQ10H_2_/T-Cho was significantly lower in the MSA group than in the Control group, even when the outlier was excluded, similar to the findings for total CoQ10 (data not shown). Raw data for each participant are summarized in Tables 2–4.

**Table 3:** Concentrations of serum CoQ10 and T-cho in patients with PD.

Patient	Sex	Age (years)	Disease duration (months)	H-Y stage/mRS	CoQ10 (nmol/l)	CoQ10H_2_ (nmol/l)	Total CoQ10 (nmol/l)	T-cho (mg/dl)	Total CoQ10/T-cho ratio
1	M	68	48	1/1	777	81	858	202	4.25
2	M	79	12	4/4	740	89	829	160	5.18
3	M	72	12	3/3	311	38	349	163	2.14
4	F	67	12	1/2	941	79	1020	205	4.98
5	M	40	24	3/2	1299	101	1400	180	7.78
6	F	66	16	3/2	544	45	589	213	2.77
7	M	75	72	3/2	520	430	950	159	5.97
8	M	53	372	3/3	276	228	504	189	2.67
9	F	66	156	4/4	205	29	234	161	1.45
10	M	41	12	1/2	310	400	710	194	3.66
11	M	56	96	3/4	678	56	734	149	4.93
12	F	78	60	2/3	717	76	793	204	3.89
13	M	67	60	3/3	749	42	791	191	4.14
14	F	65	108	3/2	496	57	553	207	2.67
15	M	67	24	2/2	273	32	305	209	1.46
16	M	47	96	2/1	792	48	840	160	5.25
17	M	74	133	3/2	352	15	367	176	2.09
18	F	50	10	3/2	1808	29	1837	229	8.02
19	M	79	12	3/2	542	35	577	140	4.12
20	M	67	4	2/2	554	22	576	146	3.93
				Mean	644.2	96.6	740.8	181.8	4.07
				SD	382.4	118.2	377.2	25.8	1.84

H-Y stage: Hoehn and Yahr stage in the “On” period.

**Table 4:** Concentrations of serum CoQ10 and T-cho in the Control group.

Patient	Sex	Age (years)	Disease	mRS	CoQ10 (nmol/l)	CoQ10H_2_ (nmol/l)	Total CoQ10 (nmol/l)	T-cho (mg/dl)	Total CoQ10/ T-cho ratio
1	F	71	Subdural hematoma	3	678	78	756	169	4.47
2	F	57	Myositis	3	1003	231	1234	174	7.09
3	F	77	NPH	2	798	55	853	124	6.86
4	F	50	Multiple sclerosis	1	420	51	471	199	2.37
5	M	61	Normal	2	1026	75	1101	193	5.70
6	F	43	Neuromyelitis optica	1	4360	267	4627	162	28.56
7	F	63	Subarachnoid hemorrhage	2	780	95	875	286	3.06
8	F	61	Multiple sclerosis	3	436	45	481	137	3.51
9	F	46	ADEM	2	469	98	567	136	4.18
10	F	76	Brain infarction	3	438	27	465	179	2.60
11	M	74	Brain infarction	3	693	28	721	175	4.12
12	M	71	Herpes zoster	2	700	39	739	156	4.74
13	M	71	Brain infarction	2	632	64	696	144	4.83
14	M	73	Epilepsy	1	873	78	951	168	5.66
15	M	63	Cervical spondylosis	3	907	87	994	239	4.16
16	M	41	Neuropathy	2	485	30	515	205	2.51
17	F	46	Multiple sclerosis	1	439	140	579	147	3.93
18	M	65	Epilepsy	1	1066	45	1111	135	8.23
				Mean	900.2	85.2	985.3	173.8	5.92
				SD	890.6	66.6	939.4	40.4	5.88

NPH, normal-pressure hydrocephalus; ADEM, acute disseminated encephalomyelitis

Note: Multiple sclerosis, neuromyelitis optica, and ADEM are collectively referred to as demyelinating diseases of the central nervous system in the text.

## Discussion

The present study showed decreased total CoQ10/T-Cho ratios in MSA compared with controls, although differences in total CoQ10 levels between groups were not significant. Since CoQ10 is distributed into lipoproteins in the liver and released into the circulation because of its hydrophobicity [12], serum CoQ10 concentrations are highly dependent on lipoprotein levels. Levels of CoQ10 normalized to T-Cho levels have thus been accepted as a better biomarker of CoQ10 deficiency than non-corrected CoQ10 levels [9]. To the best of our knowledge, this is the first study to report an association between decreased levels of serum CoQ10 and risk of MSA. Our findings suggest that serum CoQ10 has potential as a biomarker in the diagnosis of MSA. The results correspond to the facts that functionally impaired variants of the *COQ2* gene are associated with decreased levels of CoQ10 in brain tissue and an increased risk of MSA [3].

Leaving aside the obvious limitation of the small sample size that may have undermined the reliability of the results, we would like to note the bias caused by the predominance of the MSA-C subtype as a possible confounder. Phenotype distributions for MSA differ between populations. The MSA-P subtype predominates among Caucasian MSA patients, while MSA-C is more common in the Japanese population [13,14]. The percentage of patients exhibiting the MSA-C subtype in our study (88.9%) was similar to that in a previous study of Japanese patients with clinically diagnosed MSA (83.8%) [15]. According to a study of primary CoQ10 deficiency, concentrations of CoQ10 in the human brain were lowest in the cerebellum, which may thus be selectively vulnerable to CoQ10 deficiency [16]. Such findings suggest that compromised *COQ2* function and/or decreased CoQ10 concentrations may contribute to cerebellar degeneration in MSA. This idea is also supported by the fact that the ratio of patients with MSA-C to those with MSA-P was higher among carriers of deleterious *COQ2* variants than among non-carriers [3]. Considering such observations, our findings might hold true only for patients with MSA-C, and not for those with MSA-P, although the two participants with MSA-P in this study did not show marked differences in CoQ10/T-Cho ratio from the rest of the MSA group (Table 2).

In future, large-scale case-control studies including adequate numbers of MSA-P patients are needed to confirm our findings and to elucidate whether decreased ratios of serum total CoQ10/T-Cho ratio are also found in patients with MSA-P.
